# Evaluation of Clinical Characteristics and Comorbid Conditions in Pediatric Traumatic Spinal Cord Injury Patients

**DOI:** 10.7759/cureus.44512

**Published:** 2023-09-01

**Authors:** Ender Erden

**Affiliations:** 1 Physical Medicine and Rehabilitation, Hitit University Faculty of Medicine, Çorum, TUR

**Keywords:** comorbid conditions, etiology, pediatric, traumatic, spinal cord injury

## Abstract

Objective

This study aimed to examine the demographic features and the most common comorbid conditions of pediatric traumatic spinal cord injury (SCI) patients who were admitted to Ankara Physical Therapy and Rehabilitation Training and Research Hospital's inpatient rehabilitation program.

Materials and methods

The demographic features, clinical features and cormorbid conditions of 147 pediatric traumatic SCI patients (age of injury 17 and under) who received an inpatient rehabilitation program in the hospital between 2009-2017 were retrospectively examined. Patients were divided into three groups according to the lesion location (cervical, thoracic, and lumbar); and into two groups according to the age of completing the development of osteoligamentous structures in the vertebral column (group 1: ≤ 10 years and group 2: >10 years), and the evaluated data were compared.

Results

73.5% of the patients were male, the average age of injury was 13.60 ± 4.19 years, and the average duration of the disease was 11.17 ± 20.80 months. The most common etiological reason was falls from height (34.7%), and the most common level of injury was the thoracic region (49%). The most common comorbid conditions after SCI were found to be neurogenic bladder (91.2%), spasticity (41.54%), and neuropathic pain (29.3%). It was determined that neurogenic bladder was seen less in the lumbar region (p<0.001). Urinary tract infection was found more in the cervical group (p=0.004). In Group 1 (0-10 years), the median disease duration was longer, and the rate of thoracic region injury and complete injury was higher (p<0.05); in Group 2 (11-17 years), the rate of having stabilization operation after the injury was significantly higher (p<0.001).

Conclusion

It is crucial to prevent the etiological reasons in pediatric traumatic SCI patients, to treat the arising comorbid conditions in the early period, to take protective measures, and to follow up the patients regularly when necessary.

## Introduction

Spinal cord injury (SCI) is a neurological condition that often leads to permanent disabilities, with severe consequences in terms of medical, functional, economic, psychological, and social aspects. After SCI, there are vital changes and losses not only in the individual's mobility but also in many organ functions. Complications seen in patients in the acute and chronic period cause an increase in morbidity and mortality, prolongation of hospital stay, repeated admissions, a decrease in quality of life, and economic losses [1,2].

Spinal cord injury seen in the pediatric age group differs from adults in terms of the anatomical and biomechanical properties of the spine, the mechanism of injury and its outcomes, and the rehabilitation program [3]. In infants and younger children (up to the first 8 years), the fact that the head is large, neck muscles are less developed, the elasticity of internal forces is higher, and uncinate joints are not adequately developed increases the risk of cervical injury [4,5]. Pediatric SCI incidence is more common in adolescence (15-17 years) compared to childhood (0-14 years) [6]. Since SCI most often peaks in the 20-29 and 60-69 age ranges [7], there are fewer epidemiological studies involving children compared to adults. In addition, there are few publications reporting the frequency of complications during the pediatric period after pediatric SCI [7,8]. Most of the publications evaluating complications in patients with pediatric SCI provide information about evaluations made years after the injury, even during these patients' adult periods [9-12]. This situation hinders planning for patients in the pediatric age group and the development of preventive measures.

In this study, the clinical and demographic data of pediatric traumatic SCI patients who were admitted to the hospital's inpatient rehabilitation program approximately 11 months after the injury were retrospectively examined. It was aimed to present information that will allow a better evaluation of the health, economic, and political future of pediatric SCI patients. In line with the data, it was aimed to identify risk groups, determine etiological factors and common problems, and emphasize taking more effective preventive measures.

## Materials and methods

In this study, the demographic and clinical characteristics of pediatric patients with traumatic SCI (age of injury 17 and under) who received the inpatient rehabilitation program in Ankara Physical Therapy and Rehabilitation Training and Research Hospital between 2009 and 2017 were retrospectively examined from the hospital's computer or archive records. Ethical approval was obtained from the Clinical Research Ethics Committee of the Ankara Numune Training and Research Hospital (Date: 31.01.2018, Approval No: E-18-1766). The study was conducted in accordance with the principles of the Declaration of Helsinki.

Patients with non-traumatic SCI, patients with head trauma or brain injury along with SCI, patients with fractures developed in any extremity during or prior to the event and/or patients with peripheral nerve damage, and patients with communication problems were excluded from the study. A total of 147 patients were included in the study. Initial admission data were used in the evaluations.

Patients' age at injury, evaluation age, sex, duration of the event, length of hospital stay, trauma etiology, location and severity of the injury, whether they underwent an operation for spinal stabilization, functional status, comorbid conditions seen after SCI (neurogenic bladder, spasticity, neuropathic pain, pressure ulcer, urinary tract infection, deep vein thrombosis, bowel problem and others (pneumonia, contracture, vesicoureteral reflux, instability, suicidal attempt, gastrointestinal tract bleeding, post-traumatic syringomyelia)), methods of urination, and medications used were evaluated. The duration of the event was determined as the time elapsed between the injury and the patient's admission to the hospital. The medications used by the patients were classified as for neuropathic pain (gabapentin), antispasticity (tizanidine or baclofen), antimuscarinic for neurogenic bladder (oxybutynin or tolterodine), and alpha-blocker (doxazosin).

Neuropathic pain evaluation was performed based on clinical signs and symptoms. Patients aged 5-18 were included in the neuropathic pain evaluation [13].

The evaluation of neurogenic bladder was based on urodynamic examination and uroflowmetry results in patients who did not cooperate with urodynamics [14]. Urinary elimination methods used at discharge were classified as spontaneous voiding-catheterless emptying (Valsalva maneuver, triggering voiding, and anal sphincter stretching), intermittent catheterization, and permanent catheter [15].

The post-injury neurological examinations of the patients were determined using the American Spinal Injury Association Impairment Scale (AIS) classification, which evaluates motor and sensory functions in a supine position. The patient's examination is performed while the patient is lying on their back. Ten key muscles (five in the upper extremity, and five in the lower extremity) are identified for motor examination on both the right and left sides. The strength of these muscles is evaluated manually and scored between 0 and 5. For sensory examination, light touch and pin-prick (needle prick) senses are evaluated at 28 key points on the right and left sides of the body. Sensory examination is scored between 0-2 (0 none, 1 impaired, 2 normal). The neurological examination is completed by assessing the presence of anal contraction and deep anal pressure. After determining the neurological level according to the sensory and motor levels, it is examined in five classes as AIS A, B, C, D, and E. If no sensory or motor function is preserved at the sacral 4-5 level, AIS A (complete); if one of the light touch, pin-prick, or deep anal pressure senses is preserved at the sacral 4-5 level and no motor preservation is present, AIS B (sensory incomplete); if the muscle strength of less than half of the key muscles below the neurological level is three or above, AIS C (motor incomplete); if the muscle strength of half or more of these key muscles is three or above, AIS D (motor incomplete); patients with previous deficits but who have normal sensory and motor examinations are classified as AIS E [16]. Children under the age of 4 were not included in the AIS assessment [17]. 

The ambulation evaluations of the patients were performed with the functional ambulation classification (FAC), a test that evaluates lower extremity strength and dynamic balance. With FAC, ambulation is classified between 0 and 5 and consists of six items. Stage 0 identifies non-functional ambulation (cannot walk or walks with the help of two people), stage 1 identifies ambulation under the constant support and supervision of a person, stage 2 identifies ambulation with the help of a person's balance without carrying the patient's weight, stage 3 identifies ambulation dependent on observation, stage 4 identifies independent ambulation on a flat surface, and stage 5 identifies independent ambulation [18].

Patients' muscle tone was evaluated with the Modified Ashworth Scale. This scale is a scale that stages and scores spasticity in 6 degrees from 0-4 by moving the joint passively, repetitively, and rapidly while the patient is lying on their back and relaxed, and according to the resistance determined during the examination. An absence of increase in muscle tone signifies 0, a slight increase in muscle tone at the end of the range of motion signifies 1; a slight increase in muscle tone in less than half of the range of motion signifies 1+; an increase in muscle tone in most of the range of motion signifies 2, a marked increase in tone that makes passive movement difficult signifies 3; if the joint is rigid in flexion or extension, it signifies 4 [19].

Patients were divided into three groups according to the location of the lesion (cervical, thoracic, and lumbar); and into two groups according to the age of completing the development of the osteoligamentous structures in the vertebral column (group 1: ≤ 10 years and group 2: >10 years), and the evaluated data were compared [20,21].

Statistical analysis

Statistical analyses of the study were performed using Statistical Package for Social Sciences (SPSS) (Version 21, IBM Corp., Armonk, USA) and NCSS (Version 21.0.3, NCSS LLC, Kaysville, USA) programs. Descriptive statistics were presented with the mean ± standard deviation for continuous variables with normal distribution and with the median (minimum-maximum) for variables without normal distribution, along with frequencies and percentages for categorical variables. The normality of the continuous variables was tested with the Shapiro-Wilks test. Since the variables were not normally distributed in the comparison of the groups in continuous variables, the Mann-Whitney U test was used for the comparison of two groups, and the Kruskal-Wallis test was used for the comparison of more than two groups. The comparison of ratios of categorical variables was tested with the Chi-square test. In all analyses, a p < 0.05 value was considered statistically significant.

## Results

Of the patients, 108 (73.5%) were male, the mean age at injury was 13.60 ± 4.19 years, the mean age at hospital evaluation was 15.04 ± 3.80 years. The average duration of the disease was 11.17 ± 20.80 months (minimum-maximum = 0.5-240 months), and the average length of stay in our hospital was 57.71 ± 33.74 days (minimum-maximum = 1-195 days) (Table 1).

Among the etiological reasons, the top three were falling from height for 51 (34.7%) patients, motor vehicle accidents for 39 (26.5%), and gunshot wounds for 20 (13.6%). It was determined that 47 (32%) of the patients were injured at the cervical level, 72 (49%) at the thoracic level, and 29 (19%) at the lumbar level, and 132 (89.8%) underwent stabilization operations targeting the spine. 93 (65%) of the patients were AIS A (complete injury) and 132 (89.2%) were FAS 0-2 level (Table 1).

**Table 1:** Demographic and clinical features of the patients (n=147) AIS: American Spinal Injury Association Impairment Scale; FAC: Functional Ambulation Classification. The data are expressed as mean±standard deviation (minimum-maximum), or n (%).

Parameters	Results
Age at injury (years)	13.60 ± 4.19 (1.5–17)
Age at interview (years)	15.04 ± 3.80 (3–18)
Disease duration at initial admission (month)	11.17 ± 20.80 (0.5–240)
Length of stay in rehabilitation (days)	57.71 ± 33.74 (1–195)
Gender (Male)	108 (73.5)
Etiology	
Fall from height	51 (34.7)
Motor vehicle accidents	39 (26.5)
Gunshot wounds	20 (13.6)
Auto–pedestrian accidents	17 (11.6)
Diving	10 (6.8)
Stab wounds	7 (4.8)
Sports related injury	3 (2)
Level of lesion	
Cervical	47 (32)
Thoracic	72 (49)
Lumbar	28 (19)
Level of injury	
Paraplegia	100 (68)
Tetraplegia	47 (32)
AIS classification	
AIS A	93 (65)
AIS B	14 (9.8)
AIS C	23 (16.1)
AIS D	11 (7.7)
AIS E	2 (1.4)
Completeness of injury	
Complete (AIS–A)	93 (65)
Incomplete (AIS-B–E)	50 (35)
Surgical operation	132 (89.8)
FAC scala	
FAC 0–2	132 (89.8)
FAC 3–5	15 (10.2)

The most common comorbid conditions after spinal cord injury was neurogenic bladder (91.2%), followed by spasticity (41.54%), neuropathic pain (29.3%), pressure ulcer (20.4%), and urinary tract infection (11.6%). It was determined that 102 (69.4%) of the patients emptied their bladder with intermittent catheterization at the time of discharge. The bowel evaluation results of 124 patients could not be reached, and it was determined that five out of 23 evaluated patients (3.4%) had a bowel problem. The most frequently used drugs by the patients were anticholinergic, alpha-blocker, and their combination for neurogenic bladder (57.8%, 7.5%, and 6.1%, respectively) and drugs for neuropathic pain (23.8%) (Table 2). 

**Table 2:** Bladder emptying methods, post-injury complications, and drugs used by patients (n=147) The data are expressed as n, (%).

Parameters	Results
Prescribed bladder-emptying method	
Spontaneous voiding–emptying without catheter	18 (12.3)
Intermittent catheterization	102 (69.4)
Indwelling catheter	27 (18.3)
Post-injury complication	
Neurogenic bladder	134 (91.2)
Spasticity	83 (56.4)
Neuropathic pain	42 (29.3)
Pressure ulcer	30 (20.4)
Urinary tract infections	17 (11.6)
Deep venous thrombosis	6 (4.1)
Bowel problem	5 (3.4)
Pneumonia	4 (2.7)
Contracture	3 (2)
* * Vesicoureteral reflux	3 (2)
Instability	2 (1.4)
Suicidal attempt	2 (1.4)
Gastrointestinal tract bleeding	2 (1.4)
Post-traumatic syringomyelia	1 (0.7)
Medications used after injury	
Anticholinergic drugs	85 (57.8)
Anti-neuropathic drug	35 (23.8)
Anti-spasticity drug	25 (17)
Alpha-blocker drugs	11 (7.5)
Anticholinergic and alpha-blocker combination	9 (6.1)

It was determined that the neurogenic bladder was significantly less common in the lumbar group compared to the cervical and thoracic groups (p<0.001). Urinary tract infection was significantly more common in the cervical group than in the lumbar group (p=0.004). The comparison results of patients according to level groups are given in Table 3. 

**Table 3:** Comparison of patients according to the level of the injury AIS: American Spinal Injury Association Impairment Scale; FAC: Functional Ambulation Classification. The data are expressed as median (minimum-maximum), or n (%). Significant p values are written in bold. ^1^: Cervical-Thoracic; ^2^: Cervical-Lumbar; ^3^: Lumbar-Thoracic.

Parameters	Cervical n=47	Thoracic n=72	Lumbar n=28	P
Age at injury (years)	15 (1.5–17)	15 (2–17)	15 (2–17)	0.300
Age at interview (years)	17 (3–18)	16 (3–18)	17 (3–18)	0.365
Disease duration at initial admission (month)	4 (0.5–90)	4.5 (0.5–240)	2.8 (1–43)	0.626
Length of stay in rehabilitation (days)	57 (1–195)	55 (1–165)	61 (15–98)	0.434
Gender				0.079
Male	40 (85.1)	50 (69.4)	18 (64.3)	
Female	7 (14.9)	22 (30.6)	10 (35.7)	
Etiology				<0.001^2,3^
Auto–pedestrian accidents	3 (6.4)	14 (19.4)	0 (0)	
Motor vehicle accidents	14 (29.8)	21 (29.2)	4 (14.3)	
Gunshot wounds	5 (10.6)	10 (13.9)	5 (17.9)	
Fall from height	10 (21.3)	25 (34.7)	16 (57.1)	
Diving	10 (21.3)	0 (0)	0 (0)	
Stab wounds	3 (6.4)	1 (1.4)	3 (10.7)	
Sports	2 (4.3)	1 (1.4)	0 (0)	
AIS classification				<0.001^1,3^
Grade A	23 (50)	61 (87.1)	9 (33.3)	
Grade B–E	23 (50)	9 (12.9)	18 (66.7)	
Surgical operation	5 (10.6)	9 (12.5)	1 (3.6)	0.413
FAC scala				<0.001^2^^,3^
FAC 0–2	44 (93.6)	70 (97.2)	18 (64.3)	
FAC 3–5	3 (6.4)	2 (2.8)	10 (35.7)	
Prescribed bladder-emptying method				<0.001^1,2,3^
Spontaneous voiding–emptying without catheter	4 (8.5)	2 (2.8)	12 (42.9)	
Intermittent catheterization	29 (61.7)	58 (80.6)	15 (53.6)	
Indwelling catheter	14 (29.8)	12 (16.6)	1 (3.6)	
Neurogenic bladder	44 (93.6)	71 (98.6)	19 (67.9)	<0.001^2^^,3^
Urinary tract infections	11 (23.4)	6 (8.3)	0 (0)	0.004^1^^,2^
Neuropathic pain	13 (28.2)	22 (31.4)	7 (25.9)	0.847
Anti neuropathic drug	11 (23.4)	18 (25)	6 (21.4)	0.929
Anti spasticity drug	18 (38.3)	7 (9.7)	0 (0)	<0.001^1,2^
Anticholinergic drugs	28 (59.6)	46 (63.9)	11 (39.3)	0.078
Alpha-blocker drugs	4 (8.5)	3 (4.2)	11 (39.3)	0.214
Anticholinergic and alpha-blocker combination	4 (8.5)	3 (4.2)	2 (7.1)	0.608

When patients were divided into two groups according to the age of maturation of the osteoligamentous structures of the vertebral column, in group 1 (0-10 years) it was statistically significantly found that the median disease duration was longer, thoracic region injury and complete injury rates were higher (p=0.005, p=0.004, p=0.024, respectively); in group 2 (11-17 years), the rate of having stabilization surgery after injury was found to be significantly higher (p<0.001). The comparison results of patients according to age groups are given in Table 4.

**Table 4:** Comparison of patients by age group AIS: American Spinal Injury Association Impairment Scale; FAC: Functional Ambulation Classification. The data are expressed as median (minimum-maximum), or n (%). Significant p values are written in bold.

Parameters	Group 1 (0-10 years) n=27	Group 2 (11-17 years) n=120	p
Age at injury (years)	5 (1.5–10)	16 (10–17)	<0.001
Age at interview (years)	7 (3–18)	17 (11–18)	<0.001
Disease duration at initial admission (month)	12 (0.5–168)	3 (0.5–54)	0.005
Length of stay in rehabilitation (days)	43 (1–120)	56 (1–195)	0.231
Gender			0.936
Male	20 (74.1)	88 (77.3)	
Female	7 (25.9)	32 (22.7)	
Etiology			0.225
Fall from height	8 (29.6)	43 (35.8)	
Motor vehicle accidents	10 (37.0)	29 (24.2)	
Gunshot wounds	2 (7.4)	18 (15)	
Auto–pedestrian accidents	6 (22.2)	11 (9.2)	
Diving	0 (0)	10 (8.3)	
Stab wounds	1 (3.7)	6 (5.0)	
Sports	0 (0)	3 (2.5)	
Level of lesion			0.004
Cervical	4 (14.8)	43 (35.8)	
Thoracic	21 (77.8)	51 (42.5)	
Lumbar	2 (7.4)	26 (21.7)	
Completeness of injury			0.024
Complete (AIS–A)	19 (82.6)	74 (61.7)	
Incomplete (AIS-B–E)	4 (17.4)	46 (38.3)	
Surgical operation	17 (63)	115 (95.8)	<0.001
FAC scala			0.074
FAC 0–2	27 (100)	105 (87.5)	
FAC 3–5	0 (0)	15 (12.5)	
Prescribed bladder–emptying method			0.944
Spontaneous voiding–emptying without catheter	3 (11.1)	15 (12.5)	
Intermittent catheterization	20 (74.1)	82 (68.3)	
Indwelling catheter	4 (14.8)	23 (19.2)	

## Discussion

In the study, it was determined that in patients with pediatric traumatic SCI, falling from height (34.7%) was the most common etiological reason, the thoracic region (49%) was the most common injury site, and neurogenic bladder (91.2%) was the most common comorbid condition. It was observed that spasticity and the use of antispastic drugs were more common for injuries in the cervical region, complete injuries were more common in the thoracic region, and FAC 0-2 and neurogenic bladder were less common in the lumbar region. It was also determined that injuries in the thoracic region and complete injuries are more common in children under 10 years old.

It has been reported that pediatric traumatic SCI is more common in males, the average age of injury varies between 6.81-16.2 years [22-25], and the average age of patients at evaluation is 12.81 years and 13.2 years [22,23]. In a study evaluating patients with pediatric SCI from seven different countries, it was found that the length of stay for primary rehabilitation varied between 18 and 203 days [26]. Kim et al. stated that the average length of stay was the longest (32 days) in the group with SCI who received inpatient rehabilitation [27]. Another study reported that the average duration of rehabilitation for patients with pediatric SCI was 46.3 days [28]. It has been stated that the average time between pediatric SCI injury and admission for rehabilitation is 522 days, 30% of the applications are within the first 2 months after injury and 47% are within the first 6 months. At the same time, it was found that the average length of stay was 64 days in the high cervical group (C2-4), 83 days in the low cervical group (C5-8), 46 days in the thoracic group, and 39 days in the lumbosacral group [29]. In this study, the average age at injury was 13.60 years, and the average length of stay in the hospital for rehabilitation was 57.71 days. It was found that rehabilitation started 11.17 months after injury and the average length of stay was 57 days in the cervical group, 55 days in the thoracic group, and 61.5 days in the lumbar group. In addition, it was found that pediatric traumatic SCI was approximately three times more common in males than females. Most of the findings were similar to the literature. 

In studies conducted with pediatric SCI, the frequency of etiological causes has been reported at different rates. There are publications that report the most common cause in all age groups to be motor vehicle accidents [3,30], sports injuries [31], or falls from height [32]. It has been reported that pediatric SCI most commonly occurs in the thoracic region and half of the patients have complete injuries [20,33]. However, there are also studies reporting that cervical and thoracic regions can be injured at equal frequency [34], or that cervical injuries are more common [3,35]. When looking at the level of injury according to etiology, it has been reported that motor vehicle accidents most commonly cause injury in the cervical region, while falls from height most commonly cause injury in the lumbar region [36]. In the study of Erdogan and colleagues, it is stated that car accidents cause more injuries in the cervical region, motorcycle accidents in the thoracic region, falls from height and pedestrian accidents in the lumbar region, and sports injuries cause more injuries in the cervical and lumbar regions [37]. While motor vehicle accidents are generally reported as the most common cause in the literature, the most common etiological cause in this study is falling from height (34.7%), followed by motor vehicle accidents (26.5%) and firearm injuries (13.6%). Also, in the data, it was determined that motor vehicle accidents, falling from height, and diving into water cause the most injuries to the cervical region (29.8%, 21.3%, 21.3%, respectively); injuries to the thoracic region are most commonly caused by falling from height and motor vehicle accidents (34.7%, 29.2%, respectively); injuries to the lumbar region are most commonly caused by falling from height and gunshot wounds (57.1%, 17.9%, respectively). It was also found that the most common cause in the data was falling from height, the thoracic region was the most common injury site, and diving into water was more common in the cervical group than in other groups. The frequency of etiological causes and injury regions changes depending on the socioeconomic and cultural structure of the communities living in the regions from which the data is taken, as seen both in this study and in the literature. It is seen that this data should be evaluated especially when taking preventive measures to prevent pediatric SCI and that SCI is preventable. It is striking that diving into water causes high-level SCI in pediatric patients, and it is very important to inform children and parents to prevent injuries.

In a study evaluating 297 patients with pediatric traumatic SCI, it was found that motor vehicle accidents and falls were significantly more common in the 0-3 and 4-10 age groups, assault incidents were more common in the 0-3 age group, and sports events were more common in the 11-17 age group [31]. It has been stated that motor vehicle accidents are more common in children under 5 years of age [35,38], falls are more common at the age of 6-12, and hitting objects is more common at the age of 13-15 [35]. Although this study found that motor vehicle accidents were more common in the 0-10 age group and falls from height were more common in the 11-17 age group, no difference was found between the groups. However, considering that falling from height is also the most common cause of thoracic and lumbar region injuries based on the data, we can say that more caution is needed in situations that could cause falls in the 11-17 age group.

When looking at the ambulation level of patients with pediatric traumatic SCI, most patients are non-ambulatory. In a study evaluating 68 patients, it was stated that 29 of the patients were able to walk in the community at discharge (with or without using an orthosis), 23 needed a wheelchair, and 13 were able to walk at home but needed a wheelchair when going out into the community [20]. In another study, it was found that 36 of the patients with 30 pediatric traumatic and 45 non-traumatic SCI used a wheelchair [39]. In this study, 89.2% of the patients were at FAC 0-2 (non-functional or dependent on the person) level. The high rate of low FAC scores was attributed to the fact that this hospital is one of the centers that typically admit patients with severe injuries [40].

Looking at the literature, it is generally seen that complications seen in the adulthood of patients with pediatric SCI have been evaluated [9-12]. In a study where 69 patients (51 traumatic SCI and 18 non-traumatic SCI) who had SCI before the age of 18 were evaluated 27 years later, it was reported that 11.5% of the patients had normal urination and a normal bowel regimen, 37.6% had scoliosis, 66.6% had spasticity, 11.7% had syringomyelia, and 26% developed pressure ulcers [9]. Similarly, in a study conducted with 215 pediatric SCI patients with an average injury age of 13.2 years, it was stated that the most common complications seen an average of 10.3 years after the injury were urinary tract infection (72.2%), pain (66%), autonomic dysreflexia (49.1%), spasticity (46.5%), and pressure ulcers (38.3%) [41]. Vogel et al. evaluated 216 pediatric SCI patients with an average injury age of 14.1 years and an average follow-up age of 28.6 years. They found that the most common complications were urinary tract infections (74%), bowel incontinence (63%), pressure ulcers (44%), autonomic dysreflexia (42%), and respiratory complications (33%). They also noted that the most common musculoskeletal complications were pain in any region (69%), spasticity (57%), shoulder pain (48%), scoliosis (40%), hip contractures (23%), and back pain (22%). They pointed out that a young age of injury and a long duration of the disease are associated with scoliosis [11,12]. In another study, of the 31 patients with an injury age of 6.9 years and an average age of 13.9 years (range = 3-46 years) at the time of the interview, 65% of the patients described pain. Of these, 48% described nociceptive pain (musculoskeletal and visceral pain), and 19% described neuropathic pain [8]. This study is significant as it evaluated patients who received rehabilitation 11.17 months after the injury, and the comorbid conditions seen during this period were recorded. It was found that the most common comorbid condition in the patients was neurogenic bladder, followed by spasticity, neuropathic pain, pressure ulcers, and urinary tract infections.

When comparing the comorbid conditions identified with the literature information [8,9,11,12,41] where pediatric SCI patients were evaluated in their adult periods, it is seen that many comorbid conditions continue in later periods and other comorbid conditions are also added. In particular, attention should be paid to preventable problems such as pain, pressure ulcers, and urinary tract infections. It should be kept in mind that problems such as neurogenic bladder and spasticity can be seen in any period after the injury, and the compliance of the patients with the treatment can be questioned face to face or by phone at certain intervals. In this study, limited data were accessed on the neurogenic bowel, and no data were encountered regarding scoliosis. These two conditions, which affect the long-term outcomes of SCI occurring in childhood, should be given more importance. The reason why the scoliosis could not be detected was attributed to the short time passed after the event. The reason for the low number of patient data on neurogenic bowel was thought to be due to the fact that the patients did not report bowel problems. These two issues have also been very little emphasized in the literature in parallel with the data. By providing patients with the necessary information about these problems, more meaningful feedback can be obtained from patients.

In a study evaluating the long-term outcomes of pediatric SCI (both traumatic and non-traumatic), the most common bladder emptying method was found to be voluntary or involuntary bladder reflex triggering at discharge after injury (average 5.4 months), condom catheter after 10 years of injury, and intermittent catheterisation at the final evaluation (average 27 years later) [9]. In this study, it was determined that the most common method of bladder emptying at discharge was intermittent catheterization (69.4%), followed by permanent catheter (18.3%) and spontaneous voiding-voiding without catheter (12.3%). Based on these results, intermittent catheterization is a reliable bladder-emptying method in the early and late stages of injury. However, patients should be followed up at certain intervals for changes in the bladder.

When patients who had SCI before the age of nineteen were evaluated in adulthood, it was stated that the most frequently used drugs were muscle relaxants, bladder drugs, bowel drugs, analgesics, and antidepressants, in order of frequency [42]. This data is important in terms of the availability of certain drugs in the pediatric age group. In this study, it was found that the most commonly used were antimuscarinic drugs for neurogenic bladder, followed by antineuropathic drugs and antispastic drugs. No data could be reached on analgesics and antidepressant drugs.

The most significant limitation of this study is its retrospective design. The comorbid conditions reported in the study reflect the comorbid conditions seen in the post-acute and rehabilitation service, rather than the acute comorbid conditions in patients with pediatric SCI. Another limitation is the inability to access data from evaluations such as Barthel Index-Functional Independence Measure, Short Form-36, and Nottingham Health Profile, which evaluate functional status or quality of life in the rehabilitation clinic.

## Conclusions

When looking at the etiological causes in patients with pediatric traumatic SCI, it is striking that it can be prevented by family education, taking appropriate measures, and creating social awareness. It is quite important that diving into water causes cervical injuries. Due to the continuation of growth, it should be kept in mind that secondary pathologies such as scoliosis in the spine can occur years after the injury (in adulthood), patients should be informed about neurogenic bowel also in the early period, and patients should be closely followed for common comorbid conditions such as neurogenic bladder and spasticity.
